# Characteristics and outcome of elderly patients admitted for acute Cholecystitis to medical or surgical wards

**DOI:** 10.1186/s13584-020-00383-4

**Published:** 2020-08-03

**Authors:** Itamar Feldman, Lena Feldman, Dvorah S. Shapiro, Gabriel Munter, Amos M. Yinnon, Reuven Friedman

**Affiliations:** 1 Department of Geriatrics, affiliated with the Hebrew University-Hadassah Medical School, Jerusalem, Israel; 2 Division of Internal Medicine, Shaare Zedek Medical Center, affiliated with the Hebrew University-Hadassah Medical School, P.O. Box 3235, 91031 Jerusalem, Israel

**Keywords:** Cholecystitis, Medical department, Geriatric department, Outcome, Health policy

## Abstract

**Background:**

Elderly patients admitted because of acute cholecystitis are usually not operated during their initial admission and receive conservative treatment. To help formulate a new admission policy regarding elderly patients with acute cholecystitis we compared the demographic and clinical characteristics and outcome of patients > 65 with acute cholecystitis admitted to medical or surgical wards.

**Methods:**

This retrospective study included all patients > 65 years admitted for acute cholecystitis between January, 2009 and September, 2016. Data were retrieved from the electronic health records.

**Results:**

A total of 187 patients were detected, 54 (29%) in medical departments and 133 (71%) in surgical wards. The mean age (±SD) was 80 ± 7.5 and was higher among those in medical than surgical wards (84 ± 7 versus 79 ± 7, *p* <  0.05). Patients hospitalized in medical departments had more comorbidity, disability and mental impairment. However, there was no difference in mortality between the two groups, 1 (2%) and 6 (4%) respectively. Independent predictors for hospitalization in medical departments were chronic obstructive pulmonary disease (OR = 9.8, 95% C. I 1.6–59) and the Norton Scale score (NSS)(OR = 0.7, 95% C. I 0.7–0.8). Impaired mental condition was the only predictor for hospitalization > 1 week. The strongest predictor for having cholecystostomy was admission to the surgical department (OR = 14.7, 95% C. I 3.9–56.7). Linear regression showed a negative correlation between NSS and length of hospitalization (LOH; Beta = − 0.5).

**Conclusion:**

Elderly patients with acute cholecystitis who require conservative management, especially those with severe functional and mental impairment can be safely hospitalized in medical departments. Outcome was not inferior in terms of mortality and LOH. These results have practical policy implications for the placement of elderly patients with acute cholecystitis in medical rather than surgical departments.

## Introduction

Acute cholecystitis is a common clinical condition usually precipitated by cystic duct obstruction by a stone [1]. Advanced age is a risk factor for acute cholecystitis and most cases occur in older adults (50–70%) [2, 3]. These patients are usually admitted to a general surgical ward for medical treatment which includes intravenous fluids, antibiotics, restriction of oral intake and analgesics. The majority of patients treated medically experience remission of their symptoms within 2–7 days following hospitalization, although seriously ill or debilitated patients may be managed with cholecystostomy and tube drainage of the gallbladder. Cholecystectomy is the definitive treatment for cholecystitis and early cholecystectomy (i.e. operation within 72 h after 7 days of symptoms) is the preferred treatment in young patients and even in carefully selected elderly patients [4–7]. Nevertheless, many medical centers choose to treat elderly patients conservatively and delay surgery for an elective procedure. In Israel, due to logistical limitations, delayed surgery is the rule rather than the exception in many medical centers (even for young patients) [8–10]. There are several studies suggesting that conservative treatment in elderly patients is feasible and safe and is not associated with a poorer clinical outcome [9, 11, 12]. In our institution, the common practice is to discharge patients for subsequent elective laparoscopic cholecystectomy; operation during the initial admission is exceptional. Nevertheless, in other hospitals urgent or early laparoscopic cholecystectomy is the common practice [13].

Because older patients (> 65 years) often suffer from multiple comorbidities [14], it is not uncommon in our hospital to encounter older patients with acute cholecystitis in the medical or acute geriatric wards, although a prior these patients are supposed to be cared for in surgical wards.

We conducted the present study in order to formulate the most appropriate admission policy for elderly patients admitted for acute cholecystitis. Although there are data on the necessity of holistic treatment in elderly patients with surgical disease, this is the first study that evaluates management of such patients in medical wards.

We retrospectively reviewed the course and outcome of elderly patients admitted for acute cholecystitis over a 7.5 year period (2009–2016). The baseline expectation was that older patients admitted to medical departments would harbor more co-morbidities and functional disabilities [13], and therefore, would benefit from care in medical rather than in surgical departments.

## Methods

### Study design and participant selection

This study was conducted in Shaare Zedek Medical Center, a 1000-bed university affiliated, general hospital. The hospital provides all services except organ transplantations. The medical division consists of 4 medical departments and one acute geriatric department for a total of ±200 beds. The department of general surgery has ±60 beds.

We conducted a retrospective cohort review of all patients > 65 years of age with an admitting diagnosis of acute cholecystitis during a 7.5 year period (January 2009–September 2016). Patients were divided according to the department to which they were admitted after acute cholecystitis was diagnosed in the emergency department. Relevant demographic, clinical, laboratory and outcome data were compared between the two groups.

We used the Norton scale score (NSS) to evaluate the general condition of the patients. This scale, ranging from a maximal of 20 points to a minimal of 4, facilitates assessment of physical and mental conditions, activity, mobility and incontinence [15]. The NSS also enables prediction of the risk for pressure sores. The major endpoints selected for this study were (i) length of hospitalization in days (LOH), (ii) mortality, and (iii) percentage of patients treated with a cholecystostomy.

### Statistical analysis

Demographic, clinical and laboratory data were compared according to department of admission (medicine versus surgery). Categorical variables were compared using the χ2 test, and continuous variable means were compared using the Student t-test. The cut-off level for statistical analysis for difference between the groups was *p* <  0.05. Univariate linear regression was performed to explore the correlation between two continuous variables. For finding the predicting factors for outcome and admission to medical departments, a multivariate logistic regression was performed. All statistical data were calculated using the SPSS Statistic program, version 17.0 (SPSS Inc).

## Results

The study included all 187 conservatively managed patients older than 65, diagnosed with acute cholecystitis in the emergency department and admitted to either medicine (*N* = 54, 29%) or surgery (*N* = 133, 71%). The mean age of the patients was 80 ± 7. Of the total cohort, 52 (29%) were older than 85 years, 22 (13%) lived in nursing homes and the mean Norton Scale Score (NSS) was 16 ± 4. Patients hospitalized in medical departments were more likely to be older, nursing home residents, have lower NSS and more comorbidity (Table 1). The predictors for admission to medical departments were chronic obstructive pulmonary disease (COPD) (OR = 9.8, 95%CI 1.6–59, *p* <  0.05) and NSS (OR = 0.7, 95%CI 0.7–0.8, *p* <  0.05) (Table 2). Respiratory distress was more common among those hospitalized in medical departments (24% versus 5%, *p* <  0.05) (Table 3). Laboratory values were also significantly different between patients hospitalized in medical as compared to those in surgical departments (Table 3).

**Table 1:** Demographic, comorbidity and daily living characteristics of older adult patients admitted for acute cholecystitis

Characteristics	Total *n* = 187 (%)	Surgery *n* = 133 (%)	Medicine *n* = 54 (%)	p
Gender- N				0.29
Male	89 (48)	60 (45)	29 (54)	
Female	98 (52)	73 (55)	25 (46)	
Age - Mean ± SD	80 ± 7.5	79 ± 7	84 ± 7	< 0.05
Age Group - N (%)				< 0.05
65–74	38 (22)	33 (26)	5 (10)	
75–85	87 (49)	67 (52)	20 (42)	
> 85	52 (29)	29 (22)	23 (48)	
Nursing home	22 (13)	9 (7)	13 (25)	< 0.05
Morbidity – Disease N (%)				
CRF	20 (11)	12 (9)	8 (15)	0.2
CHF	27 (14)	13 (10)	14 (26)	< 0.05
DM	51 (27)	31 (23)	20 (37)	0.06
HTN	72 (38)	82 (62)	33 (61)	0.7
CVA	30 (16)	11 (8)	19 (35)	< 0.05
IHD	62 (33)	38 (29)	24 (44)	< 0.05
COPD	11 (6)	3 (2)	8 (15)	< 0.05
Norton Scale Score (NSS)	16 (4)	17 (3)	13 (4)	< 0.05
NSS Criterion^a^				
Mentally Alert	97 (66)	78 (82)	19 (37)	< 0.05
Full Mobility	54 (38)	47 (51)	7 (14)	< 0.05
Continent	87 (61)	71 (78)	16 (31)	< 0.05
Good Daily Activity	56 (42)	49 (60)	7 (14)	< 0.05

*CRF* chronic renal failure, *CHF* congestive heart failure, *DM* diabetes mellitus, *HTN* hypertension, *CVA* cerebrovascular accident, *IHD* ischemic heart disease, *COPD* chronic obstructive pulmonary disease

^a^Due to missing data, *n* = 146

**Table 2:** Multivariate factors associated with hospitalization in internal medicine/geriatric departments and clinical outcome

Risk factors	Odds ratio (OR) (95% CI)	*P*
Risk factor for admission to medical wards		
Chronic obstructive pulmonary disease	9.8 (1.6–59)	< 0.05
Norton scale score	0.7 (0.7–0.8)	< 0.05
Risk factors for mortality		
Norton scale score	0.5 (0.3–1)	0.07
Hospitalization duration > 1 week		
Impaired mental condition	3.7 (1.7–7.9)	< 0.05
Cholecystostomy		
Hospitalization in surgical ward	14.7 (3.9–56.7)	< 0.05
Chronic renal failure	3.9 (1.23–13.5)	< 0.05
Chronic obstructive pulmonary disease	16.5 (2.4–116)	
Institutional residency	6.3 (1.6–24.9)	< 0.05
Serum sodium	0.9 (0.8–1)	< 0.05
White blood count	1.1 (1.0–1.2)	< 0.05

**Table 3:** Vital signs and laboratory values of the study population

Vital signs/Laboratory values	Reference Interval	All	Surgery *n* = 133	Medicine *n* = 54	*p*
Vital Signs ±SD					
Systolic BP		143 ± 32	144 ± 32	140 ± 332	0.6
Diastolic BP		74 ± 15	74 ± 14	74 ± 17	0.9
Pulse		84 ± 18	82 ± 17	90 ± 20)	< 0.05
Respiratory Distress, N (%)		13 (10)	4 (5)	9 (24)	< 0.05
Laboratory Values ±SD					
ALT (IU/L)	0–55	126 ± 330	138 ± 369	99 ± 211	0.5
AST (IU/L)	5–34	166 ± 550	188 ± 646	112 ± 134	0.4
GGT (IU/L)	12–43	150 ± 217	122 ± 201	195 ± 251	0.1
ALP (IU/L)	38–150	147 ± 116	128 ± 87	192 ± 161	< 0.05
Total Bilirubin (mg/dL)	0.2–1.3	1.5 ± 1.3	1.5 ± 1.3	1.7 ± 1.5	0.4
LDH (IU/L)	125–220	917 ± 2214	990 ± 2596	730 ± 427	0.3
Creatinine (mg/dL)	0.52–104	1.4 ± 3.1	1.4 ± 3.3	1.3 ± 1.2	0.9
BUN (mg/dL)	9–120	24 ± 16	21 ± 11	32 ± 23	< 0.05
BUN/CRT Ratio		22 ± 8	20 ± 7	25 ± 10	< 0.05
Sodium, mEq/L	135–145	136 ± 4	136 ± 4	136 ± 6	0.9
Potassium, mEq/L	3.6–5	4.2 ± 0.6	4 ± 0.5	4.3 ± 0.8	< 0.05
WBC 10^3^/uL	3.6–10	14 ± 6	14 ± 6	14.0 ± 7	0.7
Neutrophils (%)	50–75	80 ± 12	80 ± 11	81 ± 12	0.7
Platelets 10^3^/uL	150–450	229 ± 79	224 ± 74	236 ± 92	0.7
Hemoglobin (g/dL)	12–16	13 ± 2	14 ± 2	12 ± 2	< 0.05
Amylase (IU/L)	30–125	208 ± 625	211 ± 593	201 ± 713	0.9

*BP* blood pressure, *BUN* blood urea nitrogen, *CRT* creatinine, *WBC* white blood cells

Mortality was not significantly different between the two groups (Table 4). The cause of death in all cases was sepsis. Of the cohort initially admitted to the surgical departments, six died: one died in the surgical ward and five in the medical wards (*N* = 5) or ICU (*N* = 1), where they were transferred after their initial admission to surgery. The only parameter that showed a trend toward predicting mortality was the NSS; a decrease of one point in the NSS doubled the risk of death (*P* = 0.07; Table 2). Compared to patients who survived, the NSS was lower among patients who died (16.2 vs. 9.5, *P* <  0.05). In addition, among the patients who died, two patients underwent cholecystostomy and the youngest patient was 75 years old (mean 84 ± 8 years).

**Table 4:** Clinical outcome of patients > 65 with acute cholecystitis

Clinical outcome of all patients > 65				
Outcome marker	Total *n* = 186 (%)	Surgery *n* = 133 (%)	Medicine *n* = 54 (%)	p
Days of Hospitalization (±SD)	8.9 ± 6	8.4 ± 6	10.2 ± 7	0.09
Cholecystostomy	53 (29)	45 (35)	8 (15)	< 0.05
Bacteremia	13 (7)	10 (7)	3 (6)	0.9
Death	7 (4)	6 (4)	1 (2)	0.4
Clinical outcome of the subset of patients > 75 years				
Outcome marker	Total *n* = 137 (%)	Surgery *n* = 91 (%)	Medicine *n* = 46 (%)	p
Days of Hospitalization (±SD)	9.2 ± 7	8.8 ± 7	10.0 ± 6	0.3
Cholecystostomy	41 (28)	34 (45)	7 (15)	< 0.05
Bacteremia	11 (8)	9 (9)	2 (4)	0.6
Death	7 (5)	6 (6)	1 (2)	0.7

The mean LOH was 8.9 ± 6 days and was not significantly different between the two groups (Table 4). Impaired mental condition was found to be a predictor for hospitalization longer than 1 week (OR = 3.7) (Table 2). NSS was found to have an indirect and inverse correlation with LOH (β = − 0.5).

The percentage of patients who underwent cholecystostomy was higher among patients hospitalized in surgery (45, 35%) than in medical departments (8, 14%) (*p* <  0.05). Hospitalization in surgery, nursing home residency, hyponatremia, leukocytosis and having COPD or chronic renal failure (CRF) were all found to be predictors for patients who went on to have a cholecystostomy (Table 2).

## Discussion

Traditional admission practices are changing in the current era, often driven by scarcity of beds in certain disciplines and differential reimbursement policies for various specialties. In addition, there is emerging data on geriatric co-management of elderly patients with surgical problems, such management has the advantages of shorter LOH, less mortality and lower readmission rate [16]. A combination of these and other factors has lead to a gradual increase in admission to medical departments of patients with diagnoses that previously were considered as requiring care in surgical departments (Table 5). These patients are usually elderly, with considerable functional and cognitive impairment. On the one hand, these patients are considered poor surgical candidates, while on the other hand, their multiple co-morbidities can be expected to be superiorly managed in medical departments [15, 17]. Nonetheless, both internists and surgeons questioned the clinical validity of this ensuing reality and the current study was a direct result of our quest for data. Although the current study focused on acute cholecystitis, we believe it serves as template for further studies to assess elderly patients with other, traditionally surgical diagnoses admitted to medical versus surgical departments.

**Table 5:** Several classic surgical diagnoses which could be treated in medical departments, especially in elderly patients with co-morbidities, functional and cognitive impairments who are poor surgical candidates

No.	Diagnoses
1	Acute cholecystitis
2	Ascending cholangitis
3	Pancreatitis, not gallstone-related
4	Gastro-intestinal bleeding, not life-threatening
5	Liver abscess, including gallstone-related
6	Diverticulitis

Even without an official policy, we found that 29% of elderly patients admitted for acute cholecystitis in this 7.5 year retrospective study were admitted to medical departments. As expected, they had higher rates of co-morbidities, disabilities as well as mental impairment. The percent of patients living in a nursing home in our study (12%) was much higher than that for adults over 65 years of age in Israel’s general population, which was 2% in 2015 [14]. Rates were much higher for those admitted to medicine compared to surgery (25% vs 7%, *p* <  0.05; Table 1). These data as well as the higher rates of cardiovascular, cerebrovascular and chronic respiratory disease and lower NSS indicate that the medical group had significantly more comorbidity, disability and immobility.

Patients hospitalized in medical departments presented with higher pulse rates. This could not be explained by the higher age of the population in medicine per se [18]. Possible explanations for this relative tachycardia were atrial fibrillation, the incidence of which increases with age [19], or more severe inflammatory responses among patients in the medical group. In younger patients with acute cholecystitis the mean value of liver enzymes are usually within normal range, unless the diagnosis is both acute cholecystitis and choledocholithiasis [20, 21]. In our study the mean value of all liver enzymes was above normal. This could have been due to a more serious presentation, with fatty liver disease [22] or concomitant choledocholithiasis [20], both of which are more common in the elderly [3]. Hemoglobin values, which continuously decrease in older patients [23], were found to be lower in the medical patients, who were on average older. Indeed, in our study, for the portion of the population above 75 years old, there was a negative correlation between age and hemoglobin level, as demonstrated by linear regression (beta = − 0.053, *P* = 0.06). Mean blood urea nitrogen, which also tends to increase with age [24], was found to be higher in patients admitted to medical wards. In addition, the blood urea nitrogen/creatinine ratio was higher among those in the medical group (25 vs 20), the difference implying that dehydration and hypo-perfusion were more common in this group [25].

The mortality rate of the total study population (4%) was higher than the mortality rate associated with conservative treatment of acute cholecystitis (0.8%) [11]. However, among high risk patients there are reports of rates of mortality as high as 17.5% [26]. The only parameter that showed a trend in predicting mortality was NSS (Table 2). The mean NSS among patients who died was a low 9.2, whereas the mean NSS of the entire cohort was 16. This is consistent with previous data that demonstrated the association between low NSS and mortality during hospitalization [27, 28]. In addition to a very low NSS, patients who died were very old, and five of the seven were transferred to the intensive care unit or medical department after being initially hospitalized in surgery.

The mean LOH of 8.9 ± 6 days was comparable to previous reports of elderly patients with acute cholecystitis who were managed conservatively [29]; however, it was longer than that for the general patient population (3–6 days) [30–33]. There was no statistically significant difference in LOH between the two groups.. Notably, despite the absence of statistical significance, COPD and an impaired mental condition, the two independent predictors for long hospitalization (Table 2), were more common in the medical group. There was a clear negative correlation between NSS and LOH for both groups of patients, though the correlation was weaker for patients hospitalized in medical wards.

Although the definitive treatment for acute cholecystitis is cholecystectomy, such intervention when done early has a higher rate of mortality in the elderly and other high risk patients [34, 35]. The alternative bridging treatment for high risk patients is percutaneous cholecystostomy. In our study, the cholecystostomy rate was 29%, similar to figures reported in other studies; this suggests an increasing trend of using this modality in the treatment of elderly patients diagnosed with acute cholecystitis (24–54%) [35–38].

The strongest predictor of receiving a cholecystostomy was being hospitalized in surgery (OR 15, 95% C. I 4–57), a finding which may be attributable to an increased availability of invasive procedures and a more invasive approach among surgeons. This finding suggests that physicians in medical wards should be more aware of the cholecystostomy procedure as adjunct treatment for patients with acute cholecystitis. However, not being treated with cholecystostomy was not associated with increased mortality or LOH (Table 2).

### Limitations

Our study was retrospective and this is probably its major drawback. As management of elderly patients with acute cholecystitis in medical departments has surreptitiously become an established practice in our hospital, we elected to retrospectively investigate this practice. If care in medical departments was found associated with worse outcome, this would lead to immediate reversal of the norm – and these patients would all be directed to the surgical department. However, as expected, outcome of these elderly patients in medical departments turned out to be not inferior to that in surgical departments, and was possibly associated with shorter LOH for patients with low NSS. We believe these findings facilitate embracing a new policy of admission of elderly patients with acute cholecystitis to medical departments, especially for those who are poor surgical candidates on account of multiple co-morbidities. However, in order that these data and insights lead to a generally accepted change of policy there is need for a prospective, long-term study to evaluate readmissions, morbidity and mortality and surgical interventions that may occur after the initial hospitalization.

## Conclusion

This is the first study to compare the outcomes of elderly patients with a surgical diagnosis admitted to medical and surgical wards. We found that conservative management of elderly patients with acute cholecystitis in medical departments is not inferior to treatment in surgery. Internists and geriatricians should be aware of the availability and efficacy of the percutaneous cholecystostomy for high-risk patients in medical departments. The data in this study indicate that elderly patients with acute cholecystitis, especially those with comorbidity and, functional and cognitive impairments, can and probably should be admitted to medical wards, both for the benefit of the patients themselves and because the surgical departments are not proficient in the management of these patients. However, a major concern is the already limited availability of medical beds in many hospitals, leading to frequent hallway admissions in medical departments. Our data add to other arguments to increase medical bed availability in general hospitals. Alternatively, additional medical or geriatric physicians should be made available to consult on geriatric patients in the surgical wards.

## Data Availability

Excel file is available upon request.

## Abbreviations

NSS: Norton scale score
SD: Standard deviation
COPD: Chronic obstructive pulmonary disease
LOH: Length of hospitalization
CRF: Chronic renal failure
CHF: Congestive heart failure
OR: Odds ration
CI: Confidence interval
